# Characterization of leptospira isolates from animals and humans: phylogenetic analysis identifies the prevalence of intermediate species in India

**DOI:** 10.1186/2193-1801-2-362

**Published:** 2013-07-30

**Authors:** Vinayagamurthy Balamurugan, Nidaghatta L Gangadhar, Nagalingam Mohandoss, Sushma Rahim Assadi Thirumalesh, Moushumi Dhar, Rajeswari Shome, Paramanandham Krishnamoorthy, Krishnamsetty Prabhudas, Habibur Rahman

**Affiliations:** Project Directorate on Animal Disease Monitoring and Surveillance (PD_ADMAS), Hebbal, HA Farm Post, Bengaluru, 560 024 Karnataka India

**Keywords:** Leptospira, Animals, Human, Characterization, Prevalence, Intermediate species

## Abstract

In this study, 191 culture isolates were recovered from suspected samples of animals and humans in Ellinghausen McCullough Johnson and Harris (EMJH) medium and assessed for its morphological features by dark field microscopy. Extracted DNA from individual culture was subjected to different PCR assays for identification and characterization of leptospira. Out of 99 positive leptospira cultures, 52 pathogenic leptospira isolates were characterized at species level by using partial RNA polymerase β-subunit (*rpoB)* gene sequences. Phylogenetic analysis of the nucleotide sequences revealed that 30, 8, and 14 isolates belong to *L. borgpetersenii / L. interrogans*, *L. kirschneri*, and *Leptospira* intermediate species, respectively. Based on analysis of 99 leptospira isolates, the prevalent *Leptospira* species were *L. borgpetersenii* or *L. interrogans* (30.30%), *L. kirschneri* (8%) and *Leptospira* intermediate species (14.14%) in animals and humans. To the best of authors knowledge, this is the first study to use *rpoB* gene nucleotide sequence based phylogenetic analysis to identify/detect *Leptospira* intermediate species (*L. wolffii*) in animals and humans in India. Hence, the prevalence of this species will surely emphasize the importance of consideration of *Leptospira* intermediate species and formulate a way for further studies especially in understanding the newly emerging *Leptospira* in animals and humans and to combat the problem associated with the disease conditions.

## Introduction

Leptospirosis is an important re-emerging zoonotic disease in tropical and subtropical regions of the world with various animal species acting as carriers. It is considered as an emerging global public health problem (Bharti et al. 2003), caused by different pathogenic species of leptospira and is difficult to control and eradicate in tropical developing countries like India. Hence, early detection of leptospira in the host, prompt treatment as well as creating awareness in the public are the steps that could be taken to reduce the extent of problem and the economical losses associated with it.

Diagnosis of leptospirosis is usually achieved by serological investigation particularly by microscopic agglutination test (MAT), a gold standard test. The MAT is a well proven, accepted and widely used serological test for the detection of leptospiral antibodies in animals and humans. It is very useful in the demonstration of a four-fold change in antibody titers in paired acute and convalescent sera and is of significant value in diagnosing recent infection. However, in case of animals, getting paired sera is difficult, in such case while testing of single serum in MAT, it is essential to correlate the results with clinical signs of the affected animal. Generally, very high MAT titres with a consistent clinical features are conclusive of leptospirosis.

In any particular geographical region, various leptospiral serovars are prevalent and are associated with one or more maintenance host(s) that serves as reservoir of infection. *Leptospira* spp. are traditionally classified into 29 serogroups and over 300 serovars (Levett 2001). Further, genotypic methods like DNA-DNA hybridization have identified 20 *Leptospira* spp. to date (Cerqueira and Picardeau 2009). However, antigenically related serovars are classified in two or more different species and a serogroup is often found in several species of leptospira. Serovar identification of isolates are essential to understand the prevalence of leptospira in the epidemiology of this disease, but few laboratories perform Cross Agglutination Absorption Test (CAAT) (Terpstra et al. 1985) and most isolates are therefore not identified at serovar level. Serogroups identified using the MAT have no official taxonomic status, can serve as practical purpose of grouping common antigens together. With the emergence of molecular typing methods, it has become increasingly clearer that the concept of a “serovar” is no longer fully satisfactory as it may fail to define epidemiologically important strains/isolates in an adequate manner (Cerqueira and Picardeau 2009). For example, molecular typing has been shown to give better discrimination of strains of the Grippotyphosa serogroup than serological typing (Steinen et al. 1992; Hartskeerl et al. 2004).

Phylogenetic analysis identifies three major groups of leptospira with few exceptions, based on the pathogenicity such as pathogenic, saprophytic and intermediate strains of unclear pathogenicity (Paster et al. 1991; (Matthias et al. 2008). Genes used to discriminate between species within the genus *Leptospira* include RNA polymerase β-subunit (*rpoB)* (La Scola et al. 2006), DNA gyrase subunit B *(gyrB)* (Slack et al. 2006) and leptospiral immunoglobulin-like protein (*ligB)* (Cerqueira et al. 2009). All the proposed taxonomic markers generate results consistent with those obtained with the 16S rRNA sequences in terms of clustering of the strains into three major groups.

The research activities in leptospirosis since inception of Project Directorate on Animal Disease Monitoring and Surveillance (PD_ADMAS) has led to isolation of *Leptospira* spp. from diverse animals and humans and their maintenance in the repository, development of a simple leptospira staining kit and recording of the leptospiral abortions in bovines and other animal species (Gangadhar and Rajashekar 1998; Gangadhar et al. 2006; 2008). It is imperative to know the circulating *Leptospira* species/serovars in animals and humans in different geographical locations in order to investigate the prevalence of *Leptospira* species during monitoring of the leptospirosis. This helps in appropriate use of panel of leptospira serovars in the MAT for providing proper diagnostic without false negative results. Hence, the preliminary study was undertaken to investigate the prevalence of *Leptospira* species in animals and humans using the archived cultural isolates recovered from suspected samples of animals and humans in different geographical locality of India collected during different periods up to the species level by using *rpoB gene-* based sequence and phylogenetic analysis.

## Materials and methods

### Culture media

A modified Ellinghausen McCullough Johnson and Harris (EMJH) liquid medium containing bovine serum albumin fraction V (Ellis 1986) was prepared as per standard bacteriological procedure with 200 μg of 5-fluorouracil per mL of medium.

### Clinical samples

Clinical samples such as blood, serum, urine and other materials such as kidney, tissues and fluids from aborted fetus collected from suspected animals and humans [most of cases-pyrexia of unknown origin (PUO)] from different geographical locations of India collected either by the ADMAS team or obtained through various organizations such as state animal husbandry laboratories, research institutes and from organized farms which were submitted to the laboratory for leptospira diagnosis. The details of the species from which the samples were collected and the area of origin with brief history of samples and designation of cultural isolates are presented in Table 1. The majority of clinical materials were from the states of Karnataka, Maharashtra and Gujarat. The animals and human beings from which samples were collected and submitted for diagnosis had clinical signs consistent with leptospira infection especially in humans.

**Table 1:** **Details of characterized leptospira isolates from animals and humans**

ADMAS No.	Source of sample	Brief history of samples/specimens, place, year of collection	GenBank Acc. No.
		Karnataka state	
21	Kidney	Pregnant female rat trapped at military dairy farm, Bangalore, May,1994.#	JN388643◆
121	Kidney	Female rat trapped in dairy farm at Malleshwaram, Bangalore, August, 1994.#	JN388654◆
421	Blood/Serum	Repeat breeder cow from University of Agricultural Sciences dairy farm, Bangalore, July,1995.#	JN388636♣
737	Blood	Referred human case with fever from Bowring and Lady Curzon Hospital, Bangalore, February, 1997.*	HM046992◆
840	Urine	Rat from small animal house at Institute of Animal Health and Veterinary Biological, Bangalore, October, 1997.#	JN388655◆
843	Kidney	"	JN388644◆
930	CSF	4 ½ years old girl with pyogenic mengitis, Bangalore, March,1998.*	JN388645♠
950	Blood	Referred human case aged 21years male with fever & myalgia from Victoria Hospital, Bangalore, April,1998.*	JN388646◆
966	Urine	Referred human case aged 28 years male with fever, jaundice and hematuria from Bangalore Medical college, June, 1998.*	JN388627♣
1003	Blood	Buffalo calf with pyrexia, Hassan, August, 1998,*	JN388656◆
1063	Serum	Referred human case aged 30 years male with heptorenal syndrome and leptospira serology positive from NIMHANS, Bangalore, October,1998.*	HM046989♠
1147	Serum	Referred human case aged 14 years male with icteric discolouration of eye with heptomegally from St. Marthas Hospital, Bangalore, July,1999.	JN388634♣
1178	Serum	Referred human case aged 6 years female with pyrexia for one week from, Bowring and Lady Curzon Hospital, Bangalore, October, 1999.*	HM046997♠
1175	Blood	Bullock with fever from State Breeding and Training Centre, Hassarghatta, October, 1999.*	JN388616◆
107	Kidney	Adult male rat trapped from SPCA(Animal Shelter), Kennel, Bangalore, February, 1995.#	JN388629◆
1190	Blood	Alcoholic male aged 40 years from Gowribidanur with fever for 8 days (Temperature 104°C), August, 2000.*	JN388628◆
58	Blood/ Serum	Female with pyrexia of unknown origin, February, 2010.*	JN388620◆
G202	Foetal peritoneal fluid	Aborted foetus from cow with fever, Kengeri, March, 2007.#	JN388621◆
G229	Foetal heart blood	Aborted foetus from cow with fever, Devanahalli, June, 2007.#	JN388622♣
1345	Blood	HF cross breed cow with pyrexia and diahhroea, Hosodi, Shimoga, September,2001.#	HM046993◆
1332	Urine	Urine from the bladder of rat tapped from Veterinary Hospital, Husudi area, Shimoga, September, 2001.#	JN388633♣
1590	Serum/Blood	Repeat breeder cow with pyrexia from Veterinary Hospital, Mysore Road Bangalore, March, 2004.*	HM046994◆
3233	Blood	Referred human case of adult male with weakness of the left side body for15 days without pyrexia from KIMS Hospital, Bangalore, March, 2007.*	JN388652♣
3647	Heart Blood	Rat trapped at Poultry farm, Veterinary College, Bangalore May 2009.*	JN388657◆
		**Gujarat state**	
2421	Serum	Cow with fever from Animal Disease Investigation, Ramveri, Surat, Gujarat, September, 2006.*	JN388631◆
2475	Serum	Following incidence of leptospirosis samples were collected from Deputy Director of Animal Husbandry, Ambavadi, Ahmedabad, Gujarat, October, 2006.* - Cow, Gandhinagar	JN388635◆
2757	Serum	- Cow, Maninagar	JN388639♣
2882	Serum	- Cow, Amaraiwadi	JN388642◆
2480	Serum	- Cow, Raipur	JN388647◆
2636	Serum	- Dog, Ghatlodiya	JN388648◆
2667	Serum	- Dog, Khadiya	JN388649◆
2713	Serum	- Buffalo, NavaVadaj	JN388650◆
2779	Serum	- Cow, Maninagar	JN388651♣
3334	Serum	6 ½ years old HF cross breed cow with pyrexia from Veterinary dispensary, Badekhan Chakla. Kazimaidair, Gopipur, Surat, Gujarat,August, 2007.*	JN388640♣
3360	Blood	Following incidence of leptospirosis, cow samples were collected from Paldi village, Kakanj Tal Daskroi District, Ahemadabad, Gujarat,September, 2007.*	JN388637♣
3377	Blood	"	JN388653♣
		**Maharashtra state**	
1215	Serum	Following incidence of leptospirosis, samples were collected from Disease Investigation Section (DIS),WRDDL, Aundh, Pune.* ^Thane-August, 2000;^±^Pune-August, 2005. - Goat^	HM046995♠
1194	Serum	- Dog^	JN388615♠
1856	Serum	-Cow^±^	JN388632◆
1756	Serum	-Buffalo^±^	JN388625♣
1761	Serum	-Buffalo^±^	JN388630♣
1762	Serum/Blood	-Buffalo^±^	JN388618♠
3395	Serum	Following incidence of leptospirosis, samples were collected from DIS, WRDDL, Pune, September 2007.* - Goat, Rajapur	JN388638◆
3394	Serum	- Goat, Rajapur	JN388624◆
3398	Serum	- Goat, Rajapur	JN388626◆
3397	Serum	- Goat, Pune	JN388641♣
**Other states**			
1183	Blood	Male elephant aged 70 years having fever from Department of Medicine, Veterinary college, Mannuthy Kerala, June, 2000.*	HM046990♠
1228	Serum	4 years old cow having pyrexia from District Medical office, Leptospira cell, Thodupuzha, Idukki. Kerala, June, 2001.*	JN388619◆
1256	Serum	Dog with fever and Jaundice, Guwahati, Assam, July, 2001.*	HM046991◆
G70	Blood/Serum	3 years old repeat breeding cow, Goa, January 2006.#	JN388617◆
G73	Serum	6 years old pregnant cow with pyrexia, Goa, January, 2006.#	HM046996♠
G90	Serum/Blood	Repeat breeding cow, Goa, September, 2006.#	JN388623◆

*Suspected samples received for leptospirosis diagnosis. # Randomly collected samples for leptsospira study. *Leptospira* species identified ◆ *L.interrogans or L.borgpetersenii* species, ♠ *L.krischneri* species, ♣ *Leptospira* intermediate species group.

### Isolation

For isolation of leptospira, the serum/blood/urine samples that were collected from the suspected animal were inoculated (1–2 drops) directly to the transport EMJH medium containing 500 μg of 5-fluorouracil per mL in the field level laboratory or veterinary dispensary or on the site of collection and transported to the laboratory at room temperature. Samples received from the messenger or by post either in the transport medium for isolation or submission of serum/blood samples on ice pack for diagnosis of leptospirosis, upon receipt all the samples were stored at 4°C and were further used when required. It was directly inoculated into EMJH medium for isolation. The collected urine, tissue/kidney and fetal fluid/tissues were transported to laboratory either on ice pack or on transport medium, upon receipt the particular pieces of tissues especially kidney were rinsed thoroughly in media and teased the cortex and medulla region with sterile needle using media and suspension was prepared by mincing the specimen in pestle and mortar in biosafety cabinet and inoculated into culture medium containing 5-fluorouracil for isolation of leptospira organism and to prevent contamination of other bacteria. After incubation at 30°C in the laboratory for 4–5 days, the culture were filtered through 0.2 μm membrane filter and subcultured periodically from 8 to 10 weeks. On successful isolation the cultures were stored in the semisolid EMJH medium for long-term storage as archived culture with periodical subculturing every 2 to 3 months for the viability of the organism.

In this study, 191 archived culture isolates from the samples that were collected from various hosts (Human-31; Cattle-82; Buffalo-11; Goat-11; Horse-8; Rat-25; Elephant-1; Dog-16 and water bodies-6), from different geographical locations of the country were taken for analysis to identify the isolated organisms initially as leptospira and further to characterize the pathogenic culture up to species level. These stored archived individual culture were revived again in liquid medium, for isolation of DNA for its molecular characterization along with the leptospira reference culture in liquid medium as the agarose present in the semi solid media will interfere in the pelleting of the leptospira organisms.

### Identification of the causative agent by PCR

Genomic DNA was extracted from these cultures along with reference culture serovars using QIamp DNA mini kit (Qiagen, Germany) as per manufacturer’s procedure. Initially, *Leptospira* genus-specific PCR was carried out using reported Lept 1 and Lept 2 primers as per method described by Merien et al. (1992) to differentiate the leptospira from other spirochetes. Then, 16S rRNA gene-based PCR using E1 and E2 primers was performed which would amplify the 571 bp amplicon from pathogenic leptospira (Faber et al. 2000). Then, *secY* gene (translocase)-based PCR was also employed using the G1 and G2 primer set, which would amplify the 285 bp product from pathogenic *Leptospira* spp. (Gravekamp et al. 1993). Further, pathogenic isolates were characterized by *rpoB* gene-specific PCR, which was carried out using reported *rpoB* Lept 1,900f and 2,500r primers to amplify the partial gene sequences (≈600 bp product) for species identification (La Scola et al. 2006).

### Cloning, sequencing and phylogenetic analysis

The *rpoB* PCR amplicons were purified using QIA quick gel extraction Kit (Qiagen, Germany) as per manufacturer’s protocol and cloned into pGEM-T Easy vector (Promega, USA) following standard molecular procedures using Top10F’ *E. coli* host. The recombinant plasmid DNA was isolated and confirmed by PCR and restriction endonuclease analysis. The sequencing of plasmid was carried out commercially in an automated DNA sequencer. Nucleotide (nt) sequence analysis was performed with published *rpoB* gene sequences of other *Leptospira* spp. by using NCBI BLAST (Altschul et al. 1997). Further, comparative analysis of nt sequences were carried out by using the clustal W program alignment in MEGALIGN of Lasergene 6.0 (DNASTAR Inc., USA) package and sequence identity among all intermediate species was determined.

Phylogenetic tree was constructed based on partial nucleotide sequences of *rpoB* gene by using Molecular Evolutionary Genetics Analysis (MEGA) version 4 (Tamura et al. 2007). The alignment gaps were excluded from pair wise distance estimations. The tree topologies were evaluated by using bootstrap test of phylogeny in the neighbor-joining method and the bootstrap P-values were obtained after 1000 replicates of the dataset. The tree is drawn to scale, with branch lengths in the same units as those of the evolutionary distances used to infer the phylogenetic tree. The bootstrap consensus tree inferred from 1000 replicates was taken to represent the evolutionary history of the isolates analyzed. Branches corresponding to partitions reproduced in less than 50% bootstrap replicates were collapsed. The percentage of replicate trees in which the associated isolates clustered together in the bootstrap test were shown next to the branches. The evolutionary distances were computed using the Maximum Composite Likelihood (MCL) method and were in the units of the number of base substitutions per site. For comparison, available *rpoB* gene sequences of *Leptospira* spp*.* were retrieved from GenBank, NCBI database for identifying the species of isolates, which clustered in to respective genenomospecies of the *Leptospira* genus.

## Results and discussion

Several molecular techniques have been evaluated for the identification and characterization of *Leptospira* spp. and DNA–DNA hybridization identified 20 *Leptospira* species to date with nine pathogenic *Leptospira* species (Cerqueira and Picardeau 2009). This species assignment is consistent with the results of the phylogenetic analysis based on the *rrs (16S)* gene, which codes for the 16S rRNA (Postic et al. 2000). La Scola et al. (2003) reported that analysis of a segment of *rpoB* may be useful as an initial screening test for the identification of a new isolate of leptospira using a system of similarity cut-off to define species. This technique may be useful for the detection as well as identification of leptospira in clinical or environmental samples but not for serovar determination in *L. interrogans* species (La Scola et al. 2006).

In this study, out of 191 revived cultures, 99 were found positive in leptospira genus-specific PCR. The remaining 92 cultures may be other spirochete organisms namely *Borrelia, Treponema, Leptonema,* etc., as these organism looks like a spiral under dark field microscopy examination and staining (Gangadhar and Rajashekar 1998; Gangadhar et al., 2005). Of these 99 positive leptospira cultures, 52 isolates were identified as pathogenic, when tested by different type of PCR assays as described in materials and methods. These 52 isolates were further characterized by *rpoB* gene-based nucleotide sequences and phylogenetic analyses, which revealed the prevalence of four *Leptospira* spp. {*L. borgpetersenii* or *L. interrogans* (n = 30), *L. kirschneri* (n = 8) and *Leptospira* intermediate species group (n = 14)} from animals and humans in India. *Leptospira* genus-specific PCR amplified 331bp products from the leptospira organism and different PCR assays detected 52 pathogenic organism, which also gave expected size of the PCR products from pathogenic isolates as described earlier. The remaining 47 culture isolates might be non pathogenic leptospira, which may require further study to classify these isolates using complete 16S rRNA sequencing. Further, *rpoB* gene-specific amplicons were cloned into plasmid vector, characterized and sequenced. The characterized leptospira isolates were from different species (Human-10; Cattle-21; Buffalo-4; Goat-5; Rat-7; Elephant-1; Dog-4). The partial *rpoB* gene sequences of the leptospira isolates were obtained (Table 1) after editing the primer sequences and submitted to the GenBank database (HM046989 to HM046997; JN388615 to JN388667).

By sequence analysis, the isolates showed identity with published sequences of various leptospires. *L. interrogans* was the most prevalent species among the examined samples of human and animals. In general, isolates belonging to either *L. interrogans*/*L. borgpetersenii* species showed 99 to 100% identity with reported sequences*.* Similarly, isolates belonging to *L. kirschneri* species had 98–99% identity. However, isolates belonging to intermediate species showed only 77.9% to 78.3% and 78.1 to 78.5% identity with reported sequences of *L. inadai* and *L. fainei* species, respectively. La Scola et al. (2006) described the convention of identification in practice ie., if the partial *rpoB* similarity of a test isolate or strain is lower than 92%, it should be regarded as a new species. If such a value goes above 97%, then the isolate under scrutiny should be regarded as being representative of a known species. Surprisingly, 14 isolates belonging to intermediate species group showed identities from 99.1 to 99.6% with only one leptospira strain so far reported from Thailand {*L. wolffii* serovar Khorat strain Khorat-H2 sequence-NZ_AKWX02000023} (Slack et al., 2008). This molecular characterization indicates prevalence of the *L. wolffii* intermediate species in India. In India, earlier sporadic human case reports with *L. inadai* infection and circulation of *L. inadai* in reservoir hosts have been reported (Gangadhar et al., 2000; 2008).

**Figure 1:**
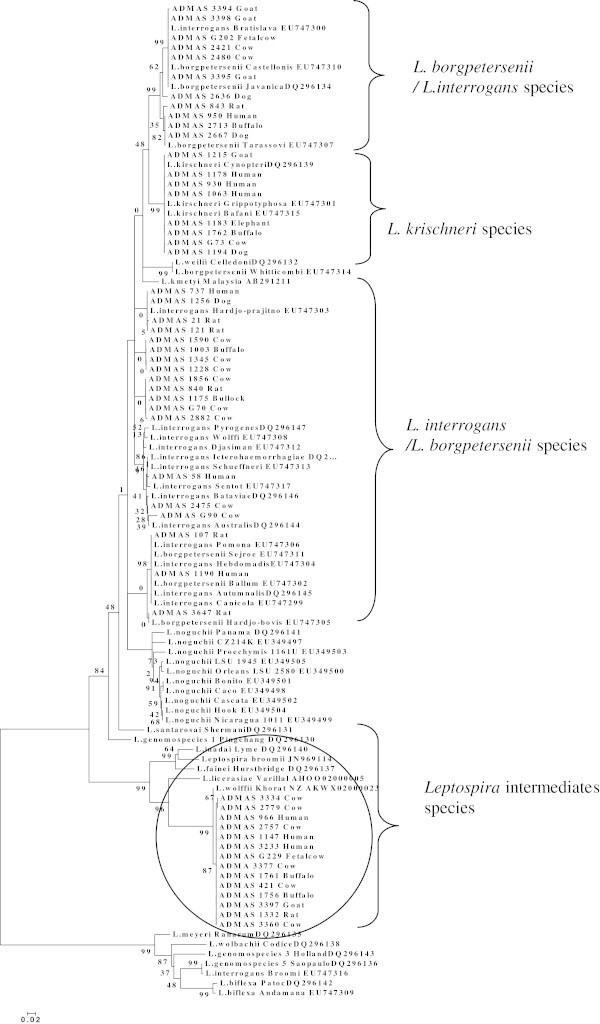
**Phylogenetic tree showing clustering of*Leptospira*constructed based on the nucleotide sequence of partial*rpoB*gene by bootstrap test of phylogeny using neighbor-joining method in MEGA-4 program.** The values of major clusters are indicated in the *node or branch* in tree, which represent the bootstrap confidence tested on using 1000 replicates of the data set. Bar represents the genetic distance i.e., number of substitutions per site.

Further, phylogenetic analysis (Figure 1) of 52 isolates based on *rpoB* gene nucleotide sequences revealed that 30 isolates belong to either *L. interrogans* or *L. borgpetersenii* species and eight belongs to *L. kirschneri* species. However, clustering of 14 leptospira isolates branched into a separate group under intermediate species (namely *L. wolffii* species). Surprisingly, when analyzing in MEGALIGN, these isolates branch into a separate group under *L. wolffii* species and showed two to three different branching pattern within this group namely 8 isolates in one branch, 4 isolates in another and 2 isolates (ADMAS No. 2779 and 3334) in other separate branches. This may clearly indicate some of the isolates, which belong to particular serovars among this species. This is to be confirmed by further analysis using the referral panel of antigen and antibodies procuring from world reference laboratory.

Based on the present study, the major prevalent pathogenic species of *Leptospira* in animals and human were *L. borgpetersenii / L. interrogans* (30.3%)*,* intermediate species (14.14%) and *L. krischneri* (8%) in India. Similarly, in the bovine population, the percentage prevalence of aforementioned species among pathogenic isolates was 56% (14/25), 36% (9/25) and 8% (2/25), respectively. In Karnataka, the prevalence rate of these species among pathogenic isolates was found to be 62.25% (15/24), 25% (6/24) and 12.5% (3/24), respectively (Table 2). The intermediate species requires further study to determine the exact serovars or new species, as recently more classification of new isolates was made for this intermediate species (Levett et al. 2006; Matthias et al. 2008). Similarly, *L. wolffii*, a potential pathogenic leptospira species detected in human, sheep and dog as intermediate species (Slack et al., 2008; Zakeri et. al., [Bibr CR30][Bibr CR31]). The presence of *L. wolffii* with 100% identity in clinical human samples and animals suspected with leptospira may provide evidence for circulation of *L. wolffii* and its role in transmission cycle within human and animal hosts in Iran (Zakeri et. al., 2010a). To establish the possible seroprevalence of this species in the population, the inclusion of intermediate species serovars representative of all the serogroups in the panel of leptospira cultural serovars besides regular serovars used in MAT for seroepidemiological studies in animals and humans as recommended earlier (Gangadhar et al. 2000).

**Table 2:** **Characterized*Leptospira*species from animals and humans in different states of India**

State	Species	***L. borgpetersenii or L. interrogans***species	***Leptospira***intermediate species	***L. krischneri***species	Total
Karnataka	Cattle	4	2	-	6
	Buffalo	1	-	-	1
	Human	4	3	3	10
	Rodents	6	1	-	7
Gujarat	Cattle	4	5	-	9
	Buffalo	1	-	-	1
	Dog	2	-	-	2
Maharashtra	Cattle	1	-	-	1
	Buffalo	-	2	1	3
	Dog	-	-	1	1
	Goat	3	1	1	5
Goa	Cattle	2	-	1	3
Kerala	Elephant	-	-	1	1
	Cattle	1	-	-	1
Assam	Dog	1	-	-	1
	**Total**	**30**	**14**	**8**	**52**

To the best of our knowledge, this is the first study to use *rpoB* gene-based phylogenetic analysis to identify or detect *Leptospira* intermediate species in animals and humans in India. Isolation and characterization of leptospires from the samples collected from different hosts, clearly suggested a possible leptospiral infection / or carrier status. Isolation of leptospires from blood samples of cows with either history of abortions or reproductive disorders does not necessarily incriminate leptospira as the etiology of the disorder but only serve as a possible indicator to the existence of the infection. Role of leptospira infection in abortions and reproductive disorders is well established by earlier workers including our earlier studies (Ellis et al. 1976; Poonacha et al. 1993; Gangadhar et al. 2008). Infected cattle are known to be maintenance host resulting in illness leading to abortion, stillbirth, infertility and mastitis or clinically normal but harbour infection and act as a potential source of infection (Higgins et al. 1980).

Leptospira and its maintenance hosts appear to undergo adaptation to their environment, and the preference and pathogenicity of these hosts can change with time and geographic region. Transmission of the infection among maintenance hosts (which remains largely asymptomatic) is efficient and the incidence of infection in humans are relatively high as they are incidental hosts or accidental hosts (which develop clinical disease). Because of the wide spectrum of animal species that serve as reservoirs/maintenance hosts, leptospirosis is considered as the most widely spread zoonotic disease.

The significance of intermediate species in public health and animal reproduction is neither clearly understood nor documented much in literature. It is imperative to identify the species prevalence as well as to know the pathogenic nature and its virulence factors, drug resistance etc. In order to understand the newly emerging leptospira in animals and humans by employing various techniques and to combat the problem associated with the disease conditions. Further studies should be carried out on characterization, identification by pulse field gel electrophoresis and hybridization techniques and pathological studies using hamster model in order to identify the exact *Leptospira* species.

The disease is of public health importance and warrants bio-safety measures in handling the organism and/or disease. Prevalence of intermediate species identified, will surely emphasize the importance of consideration of this species in India for further studies especially to understand the newly emerging leptospira in animals and humans and to combat the problem associated with the disease conditions if any. Therefore, it is important to increase attention about this disease among physicians and to strengthen laboratory capacity for its diagnosis. However, systematic random screening of the samples from different animals and at-risk personnel is required to know the exact prevalence rate in particular geographical locality, which again depends on the various epidemiological factors influencing leptospirosis occurrence and its spread. This could be useful in the selection of panel of serovars to be used in the MAT at different geographical location to provide proper diagnosis in animals and humans, which in turn will lead to development of specific diagnostics and also to determine the exact prevalence of leptospirosis in different species. Further, a large systematic epidemiology survey would be needed to define the presence and the prevalence of this pathogenic intermediate species in endemic regions of India.
